# Changes in the Splenic Melanomacrophage Centre Surface Area in Southern Bluefin Tuna (*Thunnus maccoyii*) Are Associated with Blood Fluke Infections

**DOI:** 10.3390/pathogens10010079

**Published:** 2021-01-18

**Authors:** Barbara F. Nowak, Mai Dang, Claire Webber, Lukas Neumann, Andrew Bridle, Roberto Bermudez, Daryl Evans

**Affiliations:** 1 IMAS, University of Tasmania, Launceston, TAS 7250, Australia; thi.dang@utas.edu.au (M.D.); lukas.neumann1@gmail.com (L.N.); andrew.bridle@utas.edu.au (A.B.); 2 Department of Biotechnology, Institute of Veterinary Research and Development of Central Vietnam, Km 4, 2/4 Street, Vinh Hoa, Nha Trang, Khanh Hoa 57000, Vietnam; 3 ASBTIA, Port Lincoln, SA 5606, Australia; claire@asbtia.com.au; 4 Department of Anatomy, Animal Production and Veterinary Clinical Sciences, University of Santiago de Compostela, 15705 Santiago, Spain; roberto.bermudez@usc.es; 5 Marnikol Fisheries Pty Ltd., Port Lincoln, SA 5606, Australia; dlevans@marnikol.com.au

**Keywords:** parasite, melanomacrophage centers, tuna

## Abstract

Melanomacrophage centres (MMCs) are aggregates of macrophages accumulating various pigments. They have been proposed as an indicator of fish immune response. Blood flukes are common parasites in farmed fish. Two cohorts of wild Southern Bluefin Tuna (*Thunnus maccoyi*) were examined at transfer, before treatment against blood flukes (pre-treatment) and at harvest. MMCs were assessed in histological sections using image analysis, while *Cardicola forsteri* and *Cardicola orientalis* infection severity was determined using qPCR, count of adult flukes in heart flushes and count of eggs in gill filaments. Fish from both cohorts showed the same pattern in the changes in the surface area of MMCs. The surface area of splenic MMCs increased over the ranching duration and was positively correlated to the PCR determined copy numbers of *Cardicola forsteri* ITS2 rDNA in the gills of those fish. However, the infection with blood fluke was more variable, both between cohorts and individuals within the same cohort. Eggs of blood fluke were detected in renal MMCs using histology. *Cardicola forsteri* had a higher prevalence than *Cardicola orientalis*. This study contributes to our understanding of blood fluke infections in Southern Bluefin Tuna and their interactions with MMCs.

## 1. Introduction

Melanomacrophage centres (MMCs) are groups or clusters of pigmented macrophages present in many species of poikilotherms [1]. They can be found in a range of fish organs including kidney, spleen and liver (for review see Reference [2]). Their roles include destruction and recycling of exogenous and endogenous materials, storage of iron following erythrophagocytosis, retention of resistant pathogens and antigen processing during immune response [1,3]. MMCs have been proposed as histological indicators of immune function [2].

Southern Bluefin Tuna (SBT) (*Thunnus maccoyii*) have been successfully ranched in Australia [4,5]. Wild tuna are captured in the Great Australian Bight and moved to ranching areas using towing pontoons (tows). The fish are caught from late December to March, meaning that the tows differ in fish origin as they can be from different schools of wild tuna, potentially from different geographical locations and conditions of the tow, therefore, each tow is a different cohort of fish. While overall the ranched fish are healthy, infections with blood flukes, in particular, *Cardicola forsteri* and *Cardicola orientalis*, caused mortalities in the past and require management [6]. Tuna is the final host for these blood flukes, with adult *C. forsteri* living in the heart and *C. orientalis* in blood vessels in the gills and eggs transported by blood to gills with miracidia hatching from the eggs and escaping to the external environment in search for the intermediate host (for review see Reference [7]). Presence of blood flukes, in particular, their eggs, can cause significant pathology structural changes in fish organs, which can result in mortalities [6,7].

Splenic MMCs significantly increased in size during ranching possibly due to increasing blood fluke infection, however, the infection presence or severity was not determined in that study [8]. A recent study showed a significant positive correlation between MMC size (determined as percentage surface area) and *Cardicola* spp. in the spleen for gill egg counts, and in the kidney for *C. forsteri* DNA from SBT heart [9]. However, that study focused on harvest fish only (sampling one timepoint during production cycle) and changes in MMCs during ranching season or variability between different SBT cohorts have not been determined in that study [9].

The main aim of this project was to determine the effect of ranching by sampling fish at three different time points during ranching including transfer, pre-treatment and post-treatment and the effect of the cohort of origin defined as separate tow on infection with blood fluke and splenic MMCs to increase our understanding of host response to this parasite.

## 2. Results

### 2.1. Blood Fluke Infection

For both cohorts (C1 and C2), no adult blood fluke was detected in heart flushes at transfer. Based on qPCR results, *Cardicola forsteri* was the dominant blood fluke species, whereas *Cardicola orientalis* DNA was only detected in the gills of 12 fish (13.33%). The maximum number of adult blood flukes in a heart flush was 29 (C2, pre-harvest). The infection severity varied depending on the method used, while adult blood fluke numbers in heart flushes increased from 0 at transfer to over 70% prevalence at harvest for both cohorts. Prevalence of DNA positive samples was similar at all sampling times; however, the copy number of *C. forsteri* ITS2 rDNA, measuring the parasite load in an individual fish, was greatest at harvest in both heart and gills, sometimes resulting in a statistically significant difference between pre-treatment and harvest and/or between the two cohorts (Table 1).

### 2.2. Effect of Husbandry on MMCs

**Figure 1:**
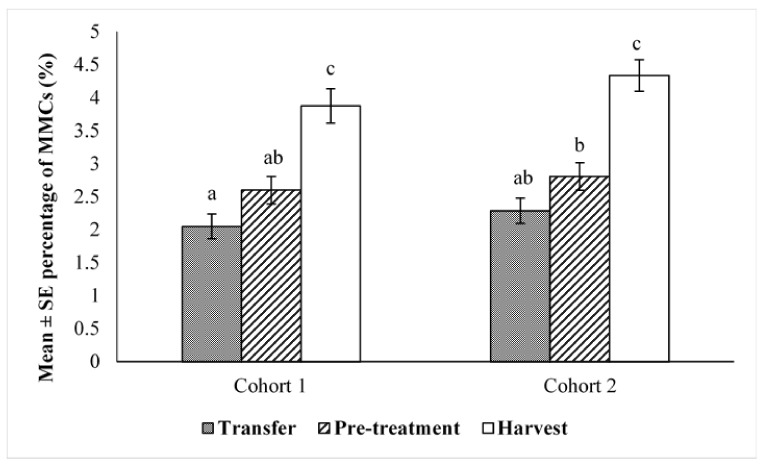
Size of splenic melanomacrophage centres (MMCs) (expressed as percentage surface area of spleen in a histological section) in Southern Bluefin Tuna (Mean ± SE). Means with different letters were significantly different (*p* < 0.05). *n* = 15 for each sampling time and cohort.

SBT from both cohorts showed the same pattern of changes in the percentage area of the spleen occupied by MMCs (Figure 1). Size of MMCs (expressed as percentage surface area) did not significantly change between transfer and pre-treatment, while it significantly increased at harvest time (*p* < 0.001). For both cohorts, the size of MMCs at harvest time was about 1.9 × higher than those at transfer time and increased 1.5 times between pre-treatment and harvest time. No significant difference was detected between the two cohorts at any sampling time (Figure 1).

### 2.3. Relationship between MMCs and Infection with Cardicola spp.

Percentage of MMCs surface area in the spleen was positively associated with copy number of *C. forsteri* ITS2 rDNA in the gills (*p* = 0.047). There were no significant relationships with other measurements of infection severity (Table 2).

**Figure 2:**
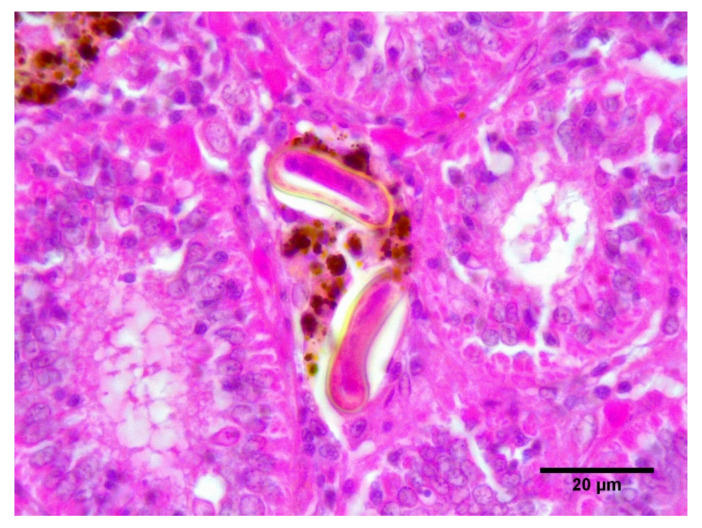
Two blood fluke eggs in a renal MMC.

In histological sections, blood fluke eggs were mainly present in the gills and heart of SBT, where they were sometimes surrounded by inflammatory infiltrate including neutrophils, monocytes and lymphocytes or by granulomatous reaction composed of epithelioid cells and fibroblasts. A few eggs were found in the renal MMCs (Figure 2) but only in three individual fish, one from C1 at transfer and two from C2, one pre-treatment and one at harvest. One fish (C2, harvest) which had 13 adult flukes in heart flush and gills positive for both *C. forsteri* and *C. orientalis* DNA had lots of large renal MMCs. No MMCs were found in the heart of any SBT. No specific histological changes related to time in ranching were observed.

The individual with more than twice larger than the average surface area of splenic MMC at transfer (C1) had blood fluke eggs present in the gills and heart and its gills were positive for *C. orientalis* DNA. Two fish from C2 pre-treatment had splenic MMC surface area above 4%, but all other fish with larger MMC surface area were from harvest (4 from C2–1.5 × larger than average and 3 from C1—almost twice as large as average MMC). All those fish which were characterized by large splenic MMCs had inflamed heart, blood fluke eggs were present either in their heart or gills and at least one other measure of blood fluke infection had positive results. However, this was not a consistent pattern as some similarly affected fish having smaller MMC.

**Figure 3:**
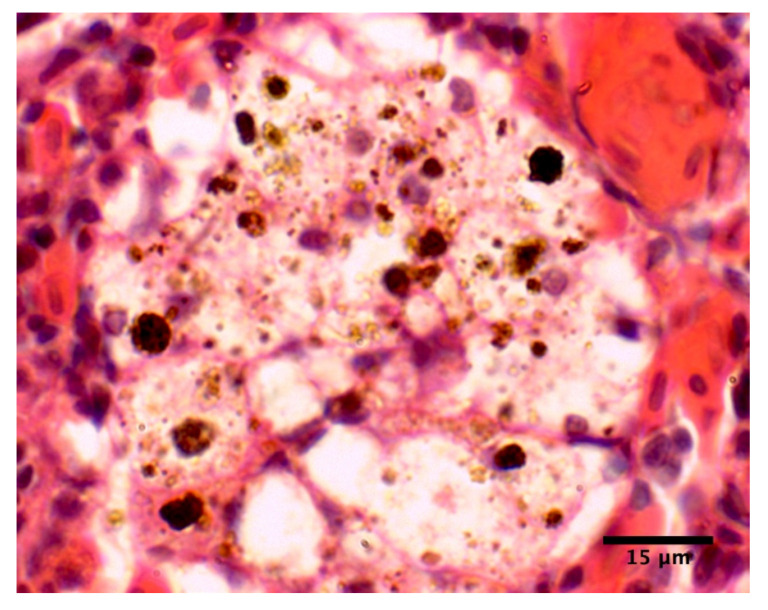
Vacuolation of MMCs in spleen.

Many splenic MMCs appeared to be vacuolated to a varying degree, mostly on the edges (Figure 3). The vacuolation seemed to be due to presence of cells with a single large most likely lipid-filled (appearing empty in histological section) not containing pigment vacuole and the nucleus in the cell periphery, possibly due to changes in cytoplasmic organization due to the vacuole formation. These cells did not appear next to other structures in the spleen such as vessels and the vacuolation was not associated with ranching time or blood fluke infection.

### 2.4. Comparison of Cohorts at Transfer

At transfer there was no significant difference between the fish from the two cohorts in weight (t = −1.08, df = 28, *p* = 0.29), length (t = −0.10, df = 28, *p* = 0.92) or severity of infection with blood fluke or metazoan ectoparasites (Table 3). Mortality to harvest was significantly greater in C2 (χ^2^ = 11.47, df = 1, *p* = 0.001, Table 4).

## 3. Discussion

Splenic MMC surface area was positively correlated to copy numbers of ITS2 rDNA *C. forsteri* in the gills. This is consistent with observations of positive correlation between splenic and renal MMC surface area and blood fluke egg count in the gills observed in ranched SBT at harvest in another season [8].

Splenic MMC size (measured as percentage surface area) was within the range previously reported for ranched SBT [7,8]. MMCs size increased significantly over ranching time from transfer to pre-treatment and harvest for both cohorts. Similar results were reported previously for a single cohort of ranched SBT [7]. This may be both (or either) due to an increase in blood fluke infection between those time points and to the increase in the fish size during ranching. A positive relationship between splenic, renal and hepatic MMC and SBT size was shown for harvest size SBT sampled in a different ranching season [8] and between splenic and renal MMC size and blood fluke infection measured as blood fluke eggs count in the gills [8] or splenic MMC size and copy number of *C. forsteri* ITS2 rDNA in the gills (present study).

While there were some differences in copy number of *C. forsteri* ITS2 rDNA in the heart between the two cohorts at harvest, overall, the infection severity was variable and there was no difference in splenic MMCs size at each sampling point. This might suggest that cohort has no effect on the splenic MMCs size, however, the cohorts were similar at transfer with regard to size, presence of macroscopic ectoparasites and blood fluke infection, and thus the lack of cohort effect result is only preliminary.

Blood fluke eggs were mainly present in gills and heart of SBT with a few eggs in renal MMCs. Blood fluke eggs were reported in hepatic MMCs in ranched SBT [8]. Eggs of another blood fluke *Cruricola lates* were trapped in hepatic and renal MMCs in barramundi, *Lates calcarifer* [10]. As usually blood fluke eggs affecting SBT would travel either from heart to gills (for *C. forsteri*) or remain in the gills (*C. orientalis*), their presence in renal or hepatic MMCs is incidental. In some species, infection with blood flukes result in the development of MMCs in the heart. For example, MMCs were present in the heart of spotted seatrout, *Cynoscion nebulosus,* infected with *Cardicola laruei* [11]. Similarly, infection by the blood fluke *Pearseonellum corventum* of leopard coral grouper, *Plectropomus leopardus* resulted in high numbers of MMCs in the heart ventricle, which was associated with the presence of blood fluke eggs [12].

There was high variability in individual infections, resulting in the lack of statistically significant differences in infection between sampling points and suggesting higher replication should be considered when designing sampling to investigate effects of different factors on blood fluke infections. Furthermore, there was some discrepancy in different measurements of the infection severity, for example, there were no adult blood flukes in heart flushes at transfer, DNA of *C. forsteri* in heart and gills and *C. orientalis* in gills was detected at that sampling point. This is not unexpected as the different tests can detect different life stages of the parasite. DNA presence corresponded with the presence of eggs in histological sections of heart and gills. *C. forsteri* was the most common species which is consistent with results since 2013 [9,13] but in contrast with reports from 2008 to 2012 [14] suggesting that there has been a change in the blood fluke dynamics infection, possibly caused by praziquantel treatment used by the industry since 2013 [9]. The increase in *C. forsteri* in gills at harvest as measured by qPCR indicates an increase in the number of eggs in the gills. This is due to reinfection after treatment and the eggs being resistant to praziquantel treatment [15] and in contrast to qPCR results for heart where there was no significant difference between transfer and harvest for each cohort. Overall, the severity of infection was within the range reported previously from ranched SBT [9,13].

In conclusion, splenic MMCs size was related to *C. forsteri* infection measured by qPCR analysis of the gills. Despite some differences between the two cohorts of SBT including heart qPCR for *C. forsteri* at harvest, the cohort had no effect on MMCs. To the best of our knowledge, this is the first report of the presence of blood fluke eggs in renal MMCs.

## 4. Materials and Methods

### 4.1. Fish

Ranched Southern Bluefin Tuna (*Thunnus maccoyii*) were sampled during ranching season between January and July 2016. The fish were from commercial pontoons from one company and were subject to standard husbandry procedures, including feeding with baitfish and treatment with praziquantel. To reduce infection by blood flukes, the fish were given praziquantel about 4 weeks after transfer (see Table 4 for the dates). Fish were treated with 15 mg/kg of praziquantel, delivered as oral treatment with a feed ration of 1.25 kg/fish on each of 2 consecutive days. Mortalities were recorded on daily bases and reported as percentage of all fish in a pontoon at harvest (Table 5).

### 4.2. Sampling Design and Sample Collection

Fish were sampled from two cohorts at three time points (transfer, pre-treatment with praziquantel and harvest). Transfer sampling occurred when the fish were transferred from the towing pontoon to a ranching pontoon, pre-treatment sampling was done a couple of days before praziquantel treatment and harvest sampling was at commercial harvest. The exact dates were dictated by commercial objectives, as a result, the cohort which was transferred earlier was harvested later (Table 4).

Fifteen fish were sampled from each cohort at each sampling point. At transfer and pre-treatment, the fish were caught on line and at harvest standard commercial harvest procedures were used. At transfer, fish weight and length were measured and the presence of ectoparasites (monogenean *Hexostoma thynni* and two species of copepods *Pseudocycnus appendiculatus* and *Euryphorus brachypterus)* determined as an additional measure of the cohort health. The characteristics of the two cohorts are presented in Table 5. Samples of gills and internal organs were fixed in 10% phosphate buffered formalin and processed for histology using routine procedures. Heart and gill samples were collected to determine the severity of infection with blood flukes using adult counts in heart flushes [16] and real-time qPCR [13]. Unless processed fresh (heart flushes), the samples were stored frozen after being fixed in RNAlater. The samples were obtained from commercially harvested fish, therefore, according to local regulations and no Ethics approval was required.

### 4.3. Severity of Infection Analysis

The severity of infection with blood flukes was determined at each sampling point. Based on our previous experience [4,13,14,16], industry records and lack of disease signs, presence of other pathogens was not investigated. The severity of infection with blood flukes was determined in several ways, including prevalence (percentage of fish affected) and intensity (infection severity/number of infected fish) and count of adult blood flukes in heart flushes and copy number of ITS2 rDNA in heart and gill samples. The copy number was calculated for *Cardicola forsteri* and *Cardicola orientalis* using species-specific primers and quantified polymerase chain reaction (qPCR) as previously described [13].

Adult blood flukes were counted in heart flushes as previously described [16].

### 4.4. Image Capture and Analysis

Percentages of MMCs surface area in a histological section were measured and calculated as the method described previously [7] using Image J software.

### 4.5. Data Analyses

A nested ANOVA (IBM SPSS statistic 22, Armonk, NY, USA)) with photo captures nested within fish within cohort and time was used to investigate if MMCs in the spleen of SBT changes over time in each group and differences between the two groups at the same time point. Because the test was robust regarding the assumption of normal distribution, so this assumption was not assessed [17,18,19]. The severity of infection was analysed using two-factor ANOVA with cohort and time as orthogonal factors and comparing between each sampling point for both cohorts. Homogeneity assumption was checked using a Levene test applying on means of MMCs quantified for each fish [20]. For comparison of the two cohorts at transfer, t-test was used. Total mortalities between the two cohorts were compared using χ^2^ test.

Possible associations between the percentage area of splenic MMCs and intensity of blood flukes in the heart and gills of SBT were assessed using Pearson correlation analysis (IBM SPSS statistic 22). *p* < 0.05 was considered statistically significant.

## Figures and Tables

**Table 1:** Severity of blood fluke infection in two cohorts (C1 and C2) of Southern Bluefin Tuna at transfer, pre-treatment and harvest. C1—Cohort 1, C2—Cohort 2 (*n* = 15 for each sampling time and cohort). Different letters show statistically significant difference (*p* < 0.05). Severity of infection was analysed using two factor ANOVA with cohort and time as orthogonal factors and all sampling points for both cohorts compared.

Infection Measure	Transfer C1 C2	Pre-Treatment C1 C2	Harvest C1 C2
Adult blood fluke heart, prevalence (%), (intensity of infection)	0 0	6.67 73 (1) (4.5)	73 80 (3) (1)
Heart *C. forsteri* qPCR, prevalence (%), (mean copy number ×10^3^, SE ×10^3^)	53 67 (114, 8) ^ab^ (9, 6) ^a^	27 40 (9,6) ^a^ (12, 7) ^a^	73 53 (915, 481) ^b^ (34, 27) ^a^
Gills *C. forsteri* qPCR, prevalence (%), (mean copy number ×10^3^, SE ×10^3^)	67 40 (47, 28) ^a^ (61, 36) ^a^	53 27 (126, 85) ^ab^ (29, 20) ^a^	100 93 (1103, 334) ^b^ (541, 495) ^ab^
Gills *C. orientalis* qPCR prevalence (%), (mean copy number ×10^3^, SE ×10^3^)	20 13 (2, 2) (7, 7)	27 13 (412, 418) (11, 12)	0 7 (6, 6)

**Table 2:** Correlation between percentage MMCs surface area of spleen and severity of infection with blood flukes in heart and gills of Southern Bluefin Tuna. *n* = 89 except *Cardicola* sp. egg where *n* = 45. * shows statistical significance. *n* = 15 for each sampling time and cohort.

Severity of Infection (Organ)	P	R
*C. forsteri* qPCR (gills) *	0.047	0.211
*C. orientalis* qPCR (gills)	0.210	−0.134
*Cardicola* spp. qPCR (gills)	0.335	0.103
*Cardicola* spp. Adult—flush and count (heart)	0.075	0.189
*C. forsteri* qPCR (heart)	0.883	−0.016

**Table 3:** Comparison of the severity of infection with parasites for the two cohorts at transfer, df = 28 for all analysis. *n* = 15 for each cohort.

Parasite	T	P
*C. forsteri* heart (qPCR)	1.57	0.13
*C. forsteri* gills (qPCR)	0.32	0.75
*C. orientalis* gills (qPCR)	0.15	0.50
*Hexostoma thynni* (count)	0.43	0.67
*Euryphorus brachypterus* (count)	1.78	0.09
*Pseudocycnus appendiculatus* (count)	0.31	0.76

**Table 4:** Sampling dates for both cohorts.

Sampling Time Point	Cohort 1	Cohort 2
Transfer	20 January 2016	18 February 2016
Pre-treatment	16 February 2016	21 March 2016
Harvest	1 July 2017	28 June 2016

**Table 5:** Characteristics of the two cohorts of Southern Bluefin Tuna. Other than transfer date, treatment date and mortality to harvest, all data are for the sampled fish only. *-significant difference between cohorts. All data for sampled fish and parasites at harvest is for *n* = 15 from each cohort.

Variable	Cohort 1	Cohort 2
Transfer date	21 January 2016	18 February 2016
Average weight at transfer (pontoon) (kg)	16.2	18.5
Average weight at transfer (sampled fish) (kg)	15.1	15.7
Average length at transfer (sampled fish) (cm)	92.3	92.4
*Hexostoma thynni* at transfer (%)	2.6	1.7
*Pseudocycnus appendiculatus* at transfer (%)	0.4	0.5
*Euryphorus brachypterus* at transfer (%)	4.3	1.1
Praziquantel treatment date	17 February 2016	22 March 2016
Mortality to harvest (%) *	3.7	4.6
Average weight at harvest (kg)	32.3	32.8

## Data Availability

The data presented in this study are available on request from the corresponding author. The data are not publicly available due to commercial reasons.
